# Single-molecular phosphorus phthalocyanine-based near-infrared-II nanoagent for photothermal antitumor therapy†

**DOI:** 10.1039/d0ra03530k

**Published:** 2020-06-12

**Authors:** Li-Na Zhou, Houhe Pan, Jing-Lan Kan, Qun Guan, Yang Zhou, Yu-Bin Dong

**Affiliations:** College of Chemistry, Chemical Engineering and Materials Science, Collaborative Innovation Centre of Functionalized Probes for Chemical Imaging in Universities of Shandong, Key Laboratory of Molecular and Nano Probes, Ministry of Education, Shandong Normal University Jinan 250014 P. R. China kanjinglan@163.com yubindong@sdnu.edu.cn; Beijing National Laboratory for Molecular Sciences (BNLMS), CAS Research/Education Center for Excellence in Molecular Sciences, Institute of Chemistry, Chinese Academy of Sciences Beijing 100190 P. R. China

## Abstract

As one of the noninvasive cancer treatments, photothermal therapy (PTT) has drawn intense attention recently. In this context, an important task is to explore novel and versatile nanoscale photothermal agents (PTAs), especially those with strong NIR-II light absorption, high photothermal conversion efficiency, good photostability and biocompatibility. Phthalocyanines (Pcs), as the second-generation photosensitizers, are a promising class of candidates for PTT due to their strong NIR absorption and high photothermal conversion efficiency. However, the poor water solubility severely limited their application as PTAs in tumor treatment. Herein, we report a molecular phosphorus phthalocyanine (P-Pc)-based nanoagent *via* incorporation of human serum albumin (HSA) under mild conditions. The obtained nanoscale P-Pc-HSA possesses excellent photothermal conversion efficiency (64.7%) upon 1064 nm light irradiation, furthermore, it can be a highly efficient NIR-II antitumor nanoagent *via* photothermal treatment (PTT), which is fully evidenced by the *in vitro* and *in vivo* experiments.

## Abstract 2

A molecular phosphorus phthalocyanine (P-Pc)-based nanoagent P-Pc-HSA, which can be a highly efficient NIR-II antitumor agent, is reported.

## Graphical abstract

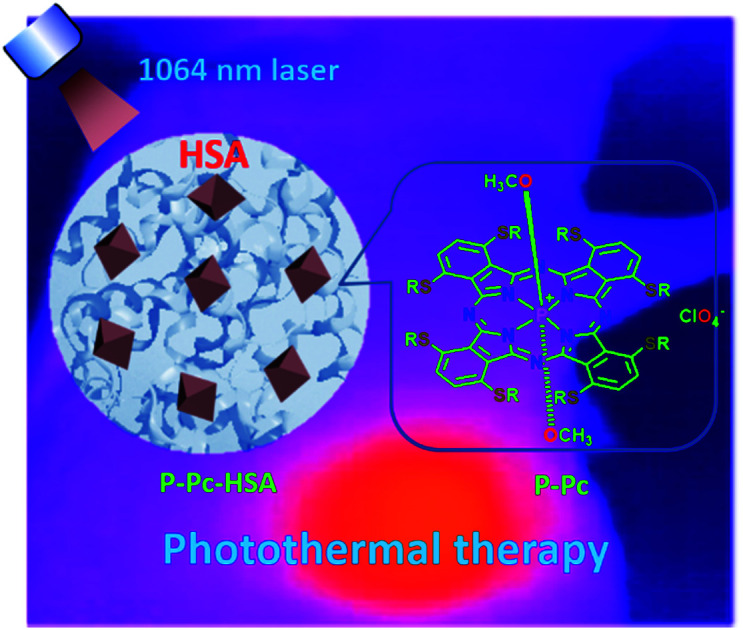

## Introduction

Photothermal therapy (PTT), as a noninvasive and potentially efficient cancer treatment, has drawn more and more attention recently.^1–5^ To date, great efforts have been devoted to studies of the nanoscale photothermal agents (PTAs) with high absorbance in the near-infrared (NIR) optical window.^6–10^ In comparison with the first near-infrared (NIR-I, 650–900 nm) window, the second near-infrared (NIR-II, 1000–1700 nm) biowindow features superior penetration depth and maximum permissible exposure (MPE) to the light, consequently, leading to better treatment efficiency.^11–15^ As it is known, an ideal PTA should possess the features such as strong NIR-II light absorption, high photothermal conversion efficiency, good photostability and biocompatibility, and easy preparation. The reported studies of the NIR-II PTAs, however, mostly focused on the inorganic and organic polymeric semiconductors,^16–20^ whereas the single small organic molecule-based nano PTAs are rarely explored.

Phthalocyanines (Pcs), as the second-generation photosensitizers, are a promising class of candidates for PTT due to their strong NIR absorption and high photothermal conversion efficiency.^21,22^ Unfortunately, the poor water solubility significantly limited their application as PTAs in tumor treatment, although some Pc-based species have been used for the photoacoustic imaging of tumor cells.^23,24^

On the other hand, human serum albumin (HSA), a major component of serum proteins, has been extensively used as a drug carrier to fabricate nanoagents due to its inherent advantages such as good biocompatibility, reduced immunogenicity, prolonged circulatory half-life, and improved pharmacokinetic properties.^25–31^ Thus, the incorporation of molecular Pcs with HSA would allow the preparation of new types of organic molecule-based nanoagents to be available.

**Fig. 1:**
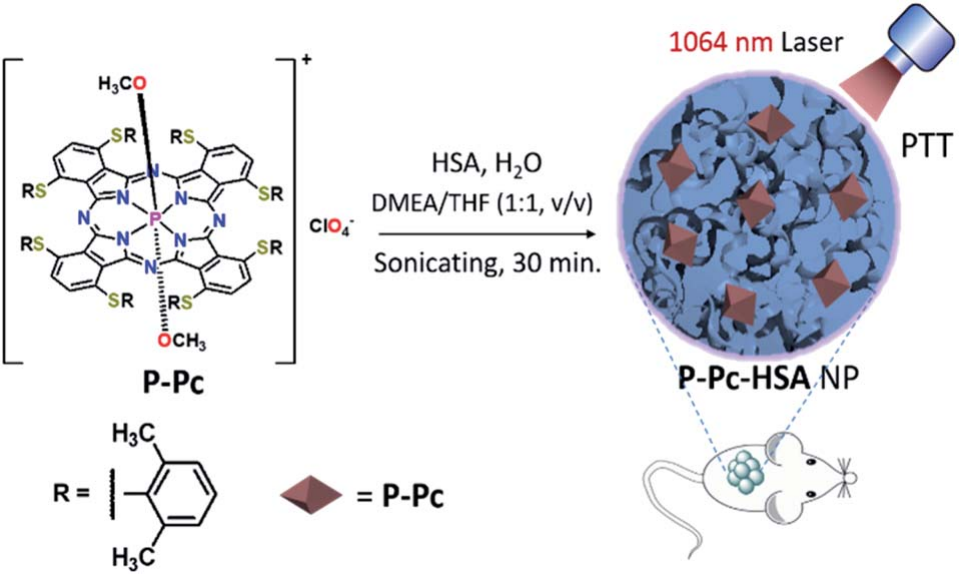
Schematic representation of preparation of P-Pc-HSA and its antitumor application *via* PTT.

In this contribution, we report, the first of its kind, an HSA-assisted and molecular phosphorus phthalocyanine (P-Pc)-based NIR-II antitumor photothermal nanoagent. The obtained nanoscale P-Pc-HSA exhibited efficient NIR-II absorption, long-term stability, good biocompatibility, low dark toxicity, and excellent photothermal conversion efficiency, which was fully evidenced by its high antitumor efficiency *via in vitro* and *in vivo* experiments (Fig. 1).

## Results and discussion

### Synthesis and characterization of P-Pc-HSA

**Fig. 2:**
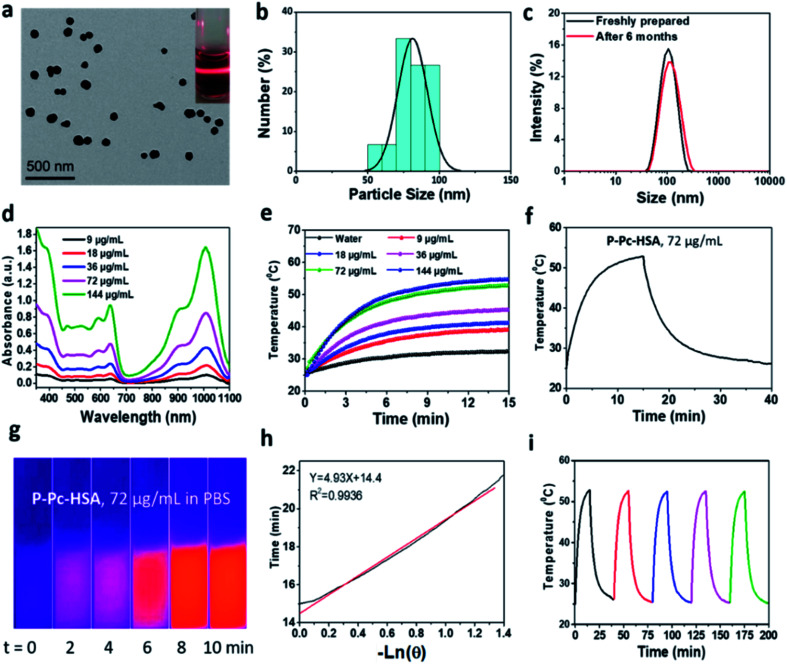
(a) TEM image of P-Pc-HSA NPs. The insets present the picture of its Tyndall phenomenon in PBS solution (pH 7.4), which further supports the TEM and DLS analysis. (b) The size distribution of P-Pc-HSA based on TEM analysis. (c) DLS size profiles of the as-synthesized P-Pc-HSA and it was allowed to stand for six months in PBS (pH 7.4) at 25 °C. (d) The absorption spectra of P-Pc-HSA in PBS (pH 7.4) at 25 °C at different concentrations. (e) Concentration-dependent temperature elevation of P-Pc-HSA in aqueous solutions under 1064 nm laser irradiation. (f) The temperature-increase of P-Pc-HSA in PBS upon 1064 nm laser irradiation. The laser was switched off at 15 min post-irradiation. (g) The thermal images of P-Pc-HSA in PBS under irradiation of 1064 nm laser for 0–10 min. (h) Plot of natural cooling time *vs.* the negative natural logarithm of the temperature driving force obtained from the cooling stage. (i) The temperature variations of P-Pc-HSA aqueous solution upon 1064 nm laser irradiation for five cycles (40 min., per cycle).

P-Pc was prepared as a dark green solid according to the reported method (Fig. S1–S6 in ESI†).^32^ As shown in Fig. 1, the P-Pc-HSA NP was prepared by quickly mixing P-Pc in *N*,*N*-dimethylethanolamine (DMEA)/tetrahydrofuran (THF) (v/v = 1 : 1) and HSA in water accompanied with sonication. After dialyzing the solution against ultrapure water to remove organic solvents, the P-Pc-HSA NPs were collected by centrifugation (see details in ESI, Fig. S7†). The P-Pc loading amount in P-Pc-HSA was determined to be 0.78 wt% based on ICP analysis. As indicated in Fig. 2a, the transmission electron microscopy (TEM) image indicated that the obtained P-Pc-HSA NPs possessed a spherical shape with the diameter of *ca.* 81.3 ± 10.3 nm (Fig. 2b). Dynamic light scattering (DLS) analysis revealed that the diameters of P-Pc-HSA NPs were centred at 110.4 ± 9.8 nm (Fig. 2c). The slightly difference in size distribution might be caused by the solvation effect depending on the different measurements. In addition, no obvious change in NP diameter was detected in different media such as H_2_O (110.4 ± 9.8 nm), PBS (117.2 ± 10.3 nm), NaCl aqueous solution (109.7 ± 9.9 nm), and DMEM (132.2 ± 12.0 nm) (Fig. S8 in ESI†). Moreover, when the PBS solution of P-Pc-HSA was allowed to stand at 4 °C for six months, no any coagulation was observed, and the measured particle size by DLS remained intact, indicating its excellent colloidal stability under physiological conditions (Fig. 2c).

The measured *ζ* potential of P-Pc-HSA in aqueous solution is *ca.* −12.8 mV (Fig. S9 in ESI†). The negatively charged surface of P-Pc-HSA NPs, together with the suitable hydrated diameter, would significantly facilitate it to accumulate at the tumor sites through the enhanced permeability and retention effect (EPR).^33^

The visible and NIR absorption displayed by P-Pc-HSA was shown in (Fig. 2d), and no change for the absorption bands was observed within the concentration range of 9–144 μg mL^−1^, implying that the P-Pc-HSA aggregates are stable based on the Lambert–Beer Law. The results of fluorescence titration analysis showed that P-Pc has a strong binding affinity with albumin by electrostatic interaction and a binding stoichiometry of 1 : 1 (see details in ESI, Fig. S10†). Notably, a strong absorption associated with the Q-band of P-Pc ranging from 900 to 1100 nm was observed. Therefore, NIR-II laser can be used as the light source for subsequent tumor treatment by PTT *in vitro* and *in vivo*.

To evaluate the photothermal properties of P-Pc-HSA, the P-Pc-HSA NPs with various P-Pc concentrations (0–144 μg mL^−1^) were irradiated under a 1064 nm laser (1.2 W cm^−2^, 15 min). As shown in Fig. 2e, the photothermal behaviour of P-Pc-HSA was concentration-dependent and laser density-dependent (Fig. S11 in ESI†). For example, when P-Pc-HSA (72 μg mL^−1^) was exposed to a 1064 nm laser (1.2 W cm^−2^) for 15 min., the system temperature increased from 25 to 52.8 °C (Δ*T* = 27.8 °C) under physiological conditions, which was further supported by its thermal imaging experiments (Fig. 2g). However, only a 7.4 °C temperature increase of pure water was detected in the absence of P-Pc-HSA under the same conditions (Fig. 2e). The photothermal conversion efficiency was calculated to be *η* = 64.7% (Fig. 2h). Notably, the photothermal conversion efficiency displayed by P-Pc-HSA herein was universally higher than those of reported phthalocyanine- and naphthalocyanine-based PTT systems (Table 1).^21,22,34–52^

**Table 1:** The reported photothermal property of the phthalocyanine (Pc) and naphthalocyanine (Nc) NPs

PTA	Material name	Wavelength/nm	*η*/%	Ref.
ZnPc	DBCO-ZnPc-LP	808	44.4	21
FePc	FePc@HSA	671	44.4	22
VONc	VONc@COF-Por	808	55.9	34
Nc	Nanomotor	808	—	35
SiNc	SiNcOH-DSPE-PEG	808	59.8	36
SiNc	SiNc-PNP	785	—	37
Pc	4OCSPC/F127	808	47.0	38
Pc	FPc	671	—	39
Pc	Pc@HSNs	730	37.1	40
MnPc	Cur-MnPc@HA	730	72.3	41
PdPc	TR-UCNS	730	54.2	42
ZnPc	SCMP	785	47.0	43
ZnPc	ZnPc-SPC	638	—	44
ZnPc	Bio-ZnPc-Pdot	808	38.2	45
ZnPc	ZnPc NPs	808	31.3	46
ZnPc	DCC@PDCZP	808	—	47
ZnPc	V-PPZ-NPs	660	—	48
ZnPc	NanoPcTB	655	—	49
ZnPc	PcS-MA	655	—	50
ZnPc	ZnPc NW	808	—	51
ZnPc	Zn_4_–H_2_Pc/DP NPs	1064	58.3	52
P-Pc	P-Pc-HSA NPs	1064	64.7	This work

Besides the high photothermal conversion efficiency, P-Pc-HSA exhibited excellent photothermal stability. As indicated in Fig. 2i, the photothermal heating efficiency of P-Pc-HSA remains intact after five laser irradiation (15 min, 1064 nm) and natural cooling cycles.

### Antitumor therapy *in vitro*

As mentioned above, P-Pc-HSA possessed excellent stability with high photothermal conversion efficiency under NIR-II light irradiation. Accordingly, the P-Pc-HSA-based PTT therapy effect on MCF-7 cells were initially tested by laser scanning confocal fluorescence microscopy.

The cellular biocompatibility and membrane permeability of P-Pc-HSA NPs was examined by cell imaging experiment, which was carried out against MCF-7 cells by confocal laser scanning microscopy (CLSM). After it (144 μg mL^−1^) incubated with MCF-7 cells for 2 h, MCF-7 cells were visualized by the green emission from P-Pc (*λ*_ex_ = 405 nm) (Fig. S12 in ESI†). We noticed that the bright green luminescence mainly located in the cytoplasm for P-Pc-HSA (Fig. S13 in ESI†), demonstrating that the P-Pc-HSA NPs could readily pass across the tumour cell membrane into the cytoplasm compared with the naked P-Pc NPs (P-Pc NPs was prepared according to the method of the synthesis of P-Pc-HSA NPs but without HSA). Thus, HSA is essential for the preparation of P-Pc nanoagent.

**Fig. 3:**
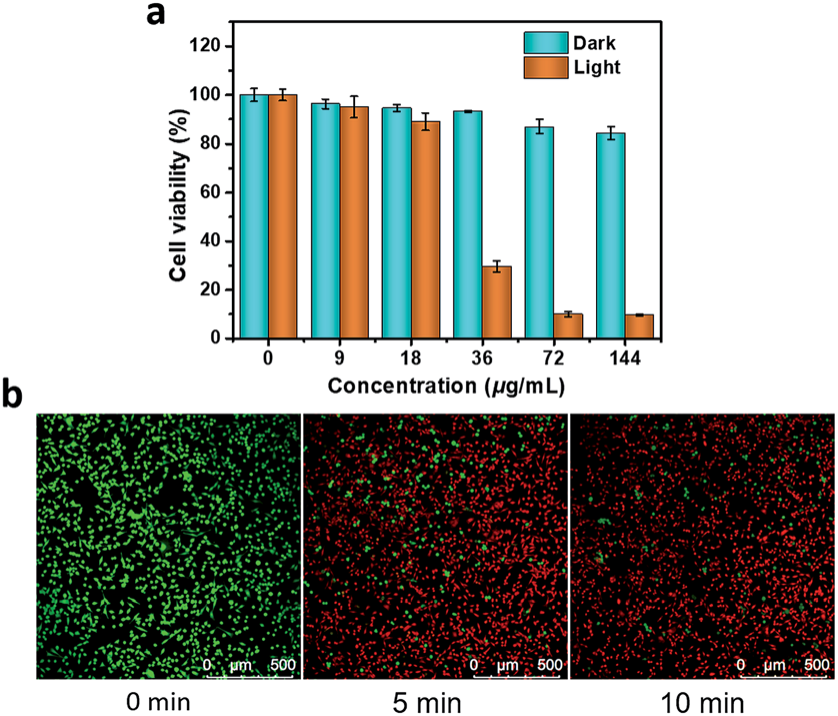
Antitumor therapy *in vitro*. (a) Cell viabilities of groups treated with PTT. The cells preincubated with P-Pc-HSA NPs (0–144 μg mL^−1^, 2 h) were exposed to a laser (1.2 W cm^−2^, 0 or 10 min) for PTT. After an additional 24 h of incubation, the cell viability was measured by a standard MTT assay. (b) Calcein-AM/PI double staining. The cells preincubated with P-Pc-HSA (72 μg mL^−1^, 2 h) were exposed to a 1064 nm laser (1.2 W cm^−2^, 0–10 min) for PTT. The cells were collected and co-stained with calcein-AM (green, living cells) and PI (red, dead cells). Scale 500 μm.

As shown in Fig. 3a, a standard 3-(4,5-dimethyl-2-thiazolyl)-2,5-diphenyl-2*H*-tetrazolium bromide (MTT) method was used to assess cell proliferation and cytotoxicity. The results showed that the cell viability remained as high as 84.6 ± 2.6% in the dark, even with a concentration as high as 144 μg mL^−1^, indicating that P-Pc-HSA possessed negligible dark toxicity and excellent biocompatibility. Even so, other types of toxicity such as genotoxicity, carcinogenicity and reproductive toxicity of P-Pc-HSA need to be further examined in the future. On the other hand, light irradiation alone also caused a negligible change in cell viability. Under 1064 nm light irradiation, however, the cell viability significantly decreased with the P-Pc-HSA concentration increasing, and the survival rates of *ca.* 29.5 ± 2.3, 9.9 ± 0.9, and 9.6 ± 0.4% were observed in the presence of 36, 72 and 144 μg mL^−1^ of P-Pc-HSA, respectively. The IC_50_ under the given treatment conditions was only 27.8 ± 0.9 μg mL^−1^ (calculated based on a logistic fit).

In addition, the prominent PTT therapeutic effect of P-Pc-HSA was further confirmed by calcein-AM/PI double staining. As shown in Fig. 3b, when a dispersion of P-Pc-HSA (72 μg mL^−1^) was used for PTT, the proportion of dead cells quickly increased with the extension of irradiation time under 1064 nm laser (1.2 W cm^−2^). The obtained result is well consistent with that of MTT assay.

### Antitumor therapy *in vivo*

Encouraged by the obtained results, we next carried out the antitumor experiments *in vivo*. The *in vivo* antitumor effect of P-Pc-HSA was evaluated by an MCF-7 xenograft model. Sixteen nude mice bearing tumors were randomly divided into four groups. Group I was the control group without any treatment. Group II was the dark group, and only P-Pc-HSA was administered (P-Pc-HSA, 50 μL, 144 μg mL^−1^, 8 min in dark). Group III was the laser group, and only light was administered (1064 nm laser, 1.2 W cm^−2^, 8 min). Group IV was the treatment group: 4 h after the intratumor injection of P-Pc-HSA (the nanoagent still retained in the tumor principally at this time), 1064 nm laser was administered to perform PTT.

**Fig. 4:**
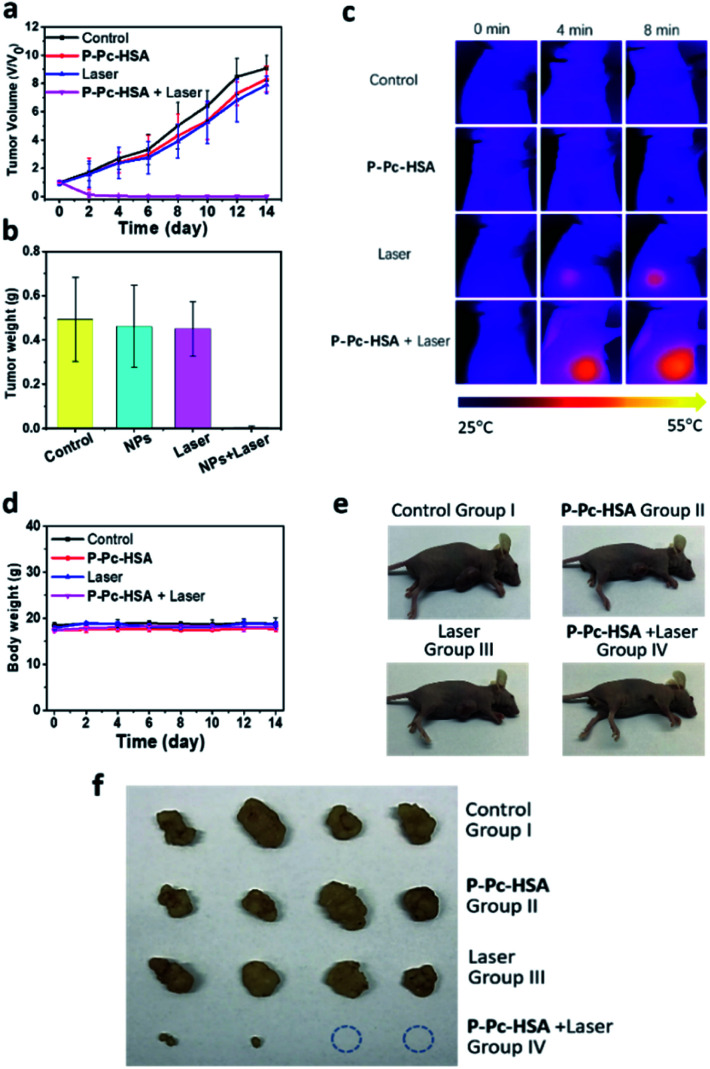
Antitumor therapy *in vivo*. Nude mice bearing MCF-7 tumors (*n* = 16) were randomly distributed into four groups when the tumor size reached *ca.* 150 mm^3^. On day 0, the nude mice were injected intratumorally, and after 4 h, laser treatment was performed on the tumor site for groups III and IV using a NIR-II laser (1.2 W cm^−2^, 8 min) for PTT. The mice continued to be fed for two weeks. The tumor volume and nude mouse weight were recorded every two days during the experiment. (a) Tumor volume of the nude mice in various groups during the treatment. (b) Tumor weight of the nude mice in various groups during the treatment. All data are presented as the mean ± SD (*n* = 4). (c) The time-dependent *in vivo* thermal images of the nude mice. (d) Body weight of the nude mice in various groups during the treatment. All data are presented as the mean ± SD (*n* = 4). (e) Photographs of the nude mice with tumor tissue obtained after treatment. (f) Photographs of the tumor tissues after dissection.

As shown in Fig. 4a and b, the tumor volume and weight increased rapidly for groups II and III, and there was no difference from the control group I. For the treatment group IV, as demonstrated by the *in vitro* experiments, PTT caused the significant temperature increase at the tumor site, which was evidenced by the thermal imaging on mice (Fig. 4c). The rapid temperature increase at tumor sites resulted in an irreversible damage, and some tumors even disappeared after 1064 nm laser irradiation, suggesting that the P-Pc-HSA nanoagent featured high *in vivo* PTT efficiency. In contrast, the rapid tumor size and weight increase in treating groups of II and III well demonstrated that only laser or P-Pc-HSA NP could only lead to the ignorable temperature increase under the given conditions. As it is shown, after 14 days treatment, there was no obvious body weight loss in all treating groups (Fig. 4d), indicating no significant systemic toxicity to the treated nude mice. The antitumor effect was further confirmed by the photographs taken before and after dissection (Fig. 4e and f).

## Experimental

### Materials and instrumentations

*p*-Toluenesulfonyl chloride was purchased from Shanghai Aladdin Biochemical Technology Co., Ltd. 2,3-Dicyanohydroquinone was purchased from Shanghai Darui Fine Chemical Co., Ltd. 2,6-Dimethylthiophenol and other organic reagents were purchased from Sahn Chemical Technology (Shanghai) Co. Phosphorous bromide was purchased from Adamas Reagent, Ltd. Sodium perchlorate, potassium carbonate, sodium sulfate and magnesium sulfate were purchased from Sinopharm Chemical Reagent Co., Ltd. HSA was purchased from Shanghai Yuanye Biotechnology Co., Ltd. All organic solvents were purchased from Sinopharm Chemical Reagent Co., Ltd. Ultra-pure water was prepared with an Aquapro System (18 MΩ).

3-(4,5-Dimethyl-2-thiazolyl)-2,5-diphenyl-2*H*-tetrazolium bromide (MTT) were purchased from Sigma-Aldrich (Shanghai) Trading Co. Ltd. Calcein O,O′-diacetate tetrakis (acetoxymethyl) ester (calcein-AM) and propidium iodide (PI) were purchased from Yeasen Biotech (Shanghai) Co., Ltd. Phosphate-Buffered Saline (PBS), Dulbecco's Phosphate-Buffered Saline (DPBS), and Fetal Bovine Serum (FBS) were purchased from Biological Industries USA, Inc. Dulbecco's Modified Eagle Medium (DMEM), Penicillin Streptomycin Mixtures (Pen-Strep), and Trypsin–EDTA Solution (0.25%) were purchased from HyClone Laboratories, Inc. Normocin was purchased from Invivogen (San Diego, CA, USA).

The 1064 nm laser (FC-1064-10W-MM) was purchased from Shanghai Xilong Optoelectronics Technology Co., Ltd. Fourier transform infrared (FT-IR) spectra were obtained in the 4000–400 cm^−1^ range using a Thermo Scientific Nicolet iS50 FT-IR spectrometer equipped with diamond attenuated total reflection (ATR) module. Each spectrum was the average of 16 scans. ^1^H NMR spectra were collected using a Bruker AVANCE 400 spectrometer. MALDI-TOF mass spectra were recorded using a Bruker BIFLEX III Ultra-High-Resolution Fourier Transform Ion Cyclotron Resonance (FT-ICR) Mass Spectrometer. Ultraviolet-visible (UV-vis) absorption spectra were recorded on a Shimadzu UV-2600 Double Beam UV-vis Spectrophotometer. Transmission electron microscope (TEM) micrographs were recorded on a Hitachi HT7700 120 kV Compact-Digital Transmission Electron Microscope. Hydrodynamic particle size and zeta potential were measured using Malvern Zetasizer Nano ZS90 System. Inductively coupled plasma (ICP) measurements were obtained on Thermo Scientific iCAP 7000 ICP-OES. Laser scanning confocal fluorescence images were captured with a Leica TCS SP8 Confocal Laser Scanning Microscopy with an objective lens (×20). Glass bottom dishes were purchased from Cellvis (Mountain View, CA, USA). Microplate assays were carried out on a Molecular Devices SpectraMax i3x Multi-Mode Microplate Detection System.

### Synthesis of P-Pc-HSA NPs

1 mL of the HSA aqueous solution (3 mg mL^−1^) was rapidly injected into 50 μL *N*,*N*-dimethylethanolamine/tetrahydrofuran (v/v = 1 : 1) solution of P-Pc (1 mg mL^−1^). Repeated the above operation for 300 times and then collected all the solutions. After sonicating the mixture for 30 minutes, the obtained aqueous dispersion was sealed in a dialysis bag (molecular weight cut-off: 3.5 kDa) and immersed in 5 L ultrapure water for 48 h, during which the water was replaced for 7 times. Then the solution of P-Pc-HSA NPs was transferred to ultra-15 mL centrifugal filters (10 kDa, Amicon Ultra-15) and centrifuged at 3900 rpm for 15 min. The generated P-Pc-HSA NPs retained in the upper tubes of the filters were readily diluted to different concentrations or re-dispersed by different solvents.

### Photostability of P-Pc-HSA NPs

Aqueous solutions of P-Pc-HSA NP (1.0 mL) with different concentrations (0, 9, 18, 36, 72, and 144 μg mL^−1^) in quartz cuvette were irradiated with a 1064 nm light at different power density between 0.3–1.5 W cm^−2^ for 15 min at 25 °C. The temperature was measured every 10 seconds using a thermocouple probe with a digital thermometer with an accuracy of 0.1 °C. The photostability of P-Pc-HSA NPs was evaluated by monitoring its photothermal effect after five cycles of laser on/off upon 1064 nm laser irradiation. Briefly, the P-Pc-HSA NPs solution (72 μg mL^−1^) was irradiated by a 1064 nm diode laser at 1.2 W cm^−2^ for 15 min (laser on), followed by naturally cooling to room temperature. The laser on and laser off cycles were repeated for five times, and the change in temperature was monitored using a thermocouple microprobe as described above.

### Photothermal conversion efficiency

To evaluate the photothermal conversion efficiency, the temperature change of the aqueous dispersion (72 μg mL^−1^) was recorded as a function of time under continuous irradiation of the 1064 nm laser with a power density of 1.2 W cm^−2^ until the solution reached a steady-state temperature.

The photothermal conversion efficiency (*η*)^16,53^ was calculated using eqn (1) described by previous reports, where *h* is the heat transfer coefficient, *A* is the surface area of the container, *T*_max_ is the equilibrium temperature, *T*_Surr_ is ambient temperature of the surroundings, Δ*T*_max_ = *T*_max_ − *T*_Surr_, *I* is incident laser power (0.942 W), and *A*_*λ*_ is the absorbance of P-Pc-HSA NPs at 1064 nm. *Q*_s_ is the heat associated with the light absorbance of the solvent, which is measured independently to be 34.5 mW using deionized water without nanoparticles.1
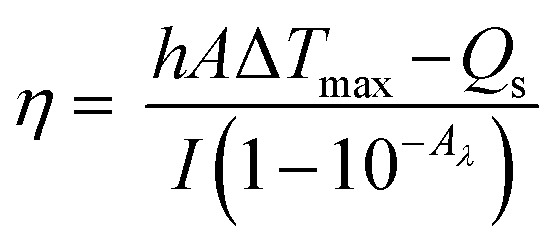


The value of *hA* is derived according to eqn (2):2
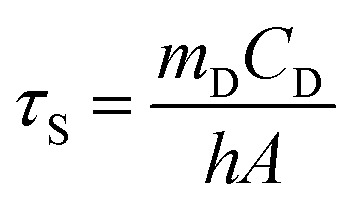
where *τ*_s_ is the time constant of sample system, mD and CD are the mass (1 g) and heat capacity (4.2 J g^−1^) of deionized water used as the solvent, respectively.

In order to obtain the *hA*, herein introduce *θ*, which is defined as the ratio of Δ*T* to Δ*T*_max_:3
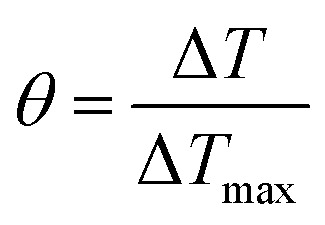
*hA* can be determined by applying the linear time data from the cooling period *versus* −ln *θ* (Fig. 2f and h). Substituting *hA* value into eqn (1), the photothermal conversion efficiency (*η*) of P-Pc-HSA NPs can be obtained.

### Cell culture and laboratory animals

The MCF-7 (human breast adenocarcinoma cell line) was provided by Stem Cell Bank, Chinese Academy of Sciences (Shanghai, P. R. China), and cultured in DMEM supplemented with FBS (10%), Normocin (50 μg mL^−1^), penicillin (100 U mL^−1^) and streptomycin (100 μg mL^−1^) in an atmosphere of CO_2_ (5 vol%) and air (95 vol%) at 37 °C.

Nude mice (BALB/c-nu, femina, aged 4 weeks, 15–20 g) were purchased from the Beijing Vital River Laboratory Animal Technology Co., Ltd. Animal experiments were reviewed and approved by the Ethics Committee of Shandong Normal University (Jinan, P. R. China). All the animal operations complied with Chinese government relevant guidelines and regulations for the care and use of experimental animals.

### Confocal laser scanning microscopy

MCF-7 cells were seeded in glass bottom dishes and incubated with NPs (144 μg mL^−1^). After 2 h, the cell culture medium was removed and cells were washed with PBS, and then imaged by a SP8 laser scanning confocal microscope. The green image of Pc was excited at 405 nm and monitored at 420–550 nm, laser intensity 20%, Smart Grain at 900 V.

### *In vitro* therapy

Cells were seeded into 96-well plates with a cell number of ∼5k cells per well and incubated overnight in a CO_2_ incubator. After removal of the culture medium, the cells were incubated with DPBS dispersion of P-Pc-HSA (100 μL, 0–144 μg mL^−1^) for 2 h in a CO_2_ incubator. The cells were exposed to 1064 nm laser (1.2 W cm^−2^, 10 min). After additional 24 h incubation, MTT (10 μL, 5 mg mL^−1^) was added to each well and incubated for additional 4 h in a CO_2_ incubator. Finally, the supernatants were removed and DMSO (100 μL) was added into each well, followed by recording the absorbance at 490 nm.

### Calcein-AM/PI double staining

MCF-7 cells were seeded in glass bottom dishes and incubated with and without P-Pc-HSA (72 μg mL^−1^, 200 μL). After 2 h, the cells were washed with PBS, and irradiated by 1064 nm (1.2 W cm^−2^) laser for different exposure time (5 min and 10 min). After that, the cells were costained with calcein-AM and propidium iodide (PI) for 10 min, washed with PBS, and then imaged by a SP8 laser scanning confocal microscope. The green images of living cells were excited by 488 nm light, and the emission wavelength range was collected at 520 ± 20 nm. The red images of dead cells were excited by 514 nm light, and the emission wavelength range was collected at 640 ± 20 nm.

### *In vivo* therapy

MCF-7 cancer cells (106 cells) suspended in DPBS (100 μL) were subcutaneously injected into the flanks of each mice to establish MCF-7 xenograft model. Length (*L*) and width (*W*) of the tumor were determined by digital calipers. The tumor volume (*V*) was calculated by the formula *V* = 1/2 × *L* × *W*^2^. When the tumor size reached ∼150 mm^3^, the nude mice bearing MCF-7 tumors (*n* = 16) were randomly distributed into four groups, as follows: (1) control, (2) P-Pc-HSA, (3) 1064 nm laser, (4) P-Pc-HSA + 1064 nm laser. After intratumoral injection, the nude mice were feeding for 4 h, and for the treatment group, light treatment was performed on the tumor site. The temperature changes at the tumor sites were recorded by an infrared thermal camera during the irradiation, and the infrared thermal images were taken at the same time. The mice continued to be fed for two weeks. The tumor volume and nude mouse weight were recorded every two days during the experiment.

## Conclusions

In summary, a P-Pc-HSA nanoagent was successfully prepared *via* incorporation of molecular P-Pc with HSA under mild conditions. The obtained P-Pc-HSA featured a strong NIR-II adsorption, excellent colloidal stability and photostability under physiological conditions. Notably, it exhibited remarkable photothermal property with high photothermal conversion efficiency up to 64.7% upon 1064 nm light irradiation. Its NIR-II light induced *in vitro* and *in vivo* PTT experiments revealed that P-Pc-HSA possessed an excellent antitumor PTT effect. These advantages imply that the P-Pc-HSA could be potentially as a highly efficient, low-toxicity and long-term small organic molecule-based nanoagent for PTT cancer treatment. We expect that our strategy herein could provide an alternative way to construction of many other types of phthalocyanine-based photothermal nanoagents for tumor treatment in the NIR-II region.

## Conflicts of interest

There are no conflicts to declare.

## Supplementary Material

RA-010-D0RA03530K-s001
