# Supplementary material for: Single-molecular phosphorus phthalocyanine-based near-infrared-II nanoagent for photothermal antitumor therapy
Source: RSC Adv. 2020 Jun 12;10(38):22656–62. doi: 10.1039/d0ra03530k (PMC9054689; doi:10.1039/d0ra03530k)
Supplement: RA-010-D0RA03530K-s001 [file RA-010-D0RA03530K-s001.pdf]

Electronic Supplementary Material (ESI) for RSC Advances.
This journal is © The Royal Society of Chemistry 2020

Supporting Information for

Single-molecular phosphorus phthalocyanine-based near-infrared-II nanoagent for photothermal antitumor therapy

Li-Na Zhou,[a] Houhe Pan,[b] Jing-Lan Kan,*[a] Qun Guan,[a] Yang Zhou,[a] and Yu-Bin Dong*[a]

[a] College of Chemistry, Chemical Engineering and Materials Science, Collaborative Innovation Centre of Functionalized Probes for Chemical Imaging in Universities of Shandong, Key Laboratory of Molecular and Nano Probes, Ministry of Education, Shandong Normal University, Jinan 250014, P. R. China.

[b] Beijing National Laboratory for Molecular Sciences (BNLMS), CAS Research/Education Center for Excellence in Molecular Sciences, Institute of chemistry, Chinese Academy of Sciences, Beijing 100190, P. R. China.

*Correspondence: kanjinglan@163.com, yubindong@sdnu.edu.cn

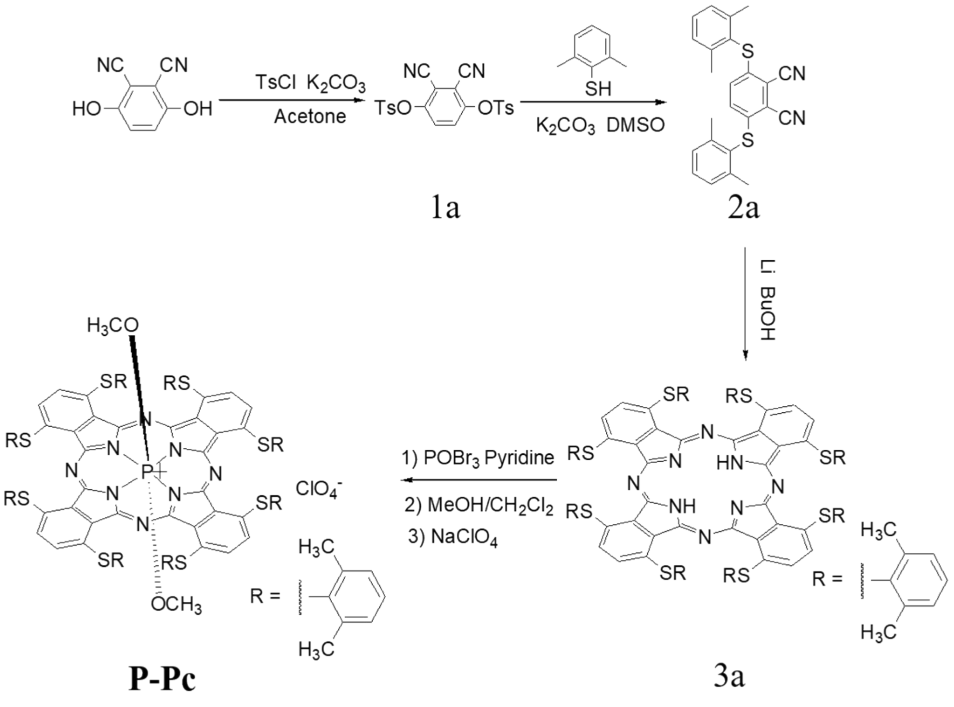

Fig. S1. Synthesis of **P-Pc**.

3,6-Bis(4′-methylphenylsulfonyloxy)phthalonitrile (1a): *p*-Toluenesulfonyl chloride (5.20 g, 27 mmol) was added to a mixture of 2,3-dicyanohydroquinone (2.00 g, 12.49 mmol) and potassium carbonate (6.90 g, 50 mmol) in acetone (15 mL). The mixture was heated to reflux for 2 h. Thin layer chromatography (TLC) was performed to determine the consumption of compound 2,3-dicyanohydroquinone. The mixture was cooled to room temperature, poured into water (40 mL)

and stirred for 1 h. The light brown product was filtered and oven dried. Yield: 5.29 g (90.4%). IR [ν_{max}/cm^{-1}]: 3432, 3239, 3085, 2243, 2226 (CN), 1504, 1449, 1315, 1279, 1204, 1174, 1142, 1021, 1004, 979, 934, 847, 749, 694, 638, 614.

3,6-Bis(phenylthio)phthalonitrile (2a): 1a (2.34 g, 5 mmol) and potassium carbonate (2.80 g, 20 mmol) were added to a solution of 2,6-dimethylthiophenol (4.40 mg, 30 mmol) in DMSO. After stirred for 14 h at room temperature, the reaction was quenched with water. The mixture was extracted with $CHCl_3$ and washed with 5% $NaCO_3$ solution and water. The organic layer was dried over $MgSO_4$ and concentrated in vacuo. The product was purified by silica gel column chromatography ($CHCl_3$) followed by recrystallization with MeOH. Title compound was obtained (200 mg, 10%) as a pale yellow powder. ^{1}H-NMR (400 MHz, $CDCl_3$) δ (ppm): 7.25 (d, 2H), 7.17 (d, 4H), 6.40 (s, 2H), 2.36 (s, 12H).

***α*-(2,6-Dimethylphenylthio)$_8$PcH$_2$ (3a)**: **2a** (103 mg, 0.3 mmol) was added to a solution of lithium (14 mg, 2.0 mmol) in 1mL of *n*-butanol and heated under reflux for 2 h. The mixture was purified by alumina gel column chromatography ($CHCl_3$). The title compound was obtained (63.8 mg, 62%) as a dark red powder. MALDI-TOF mass calculated for $C_{96}H_{82}N_8S_8$ [M$^+$]: 1602.442; found: 1602.035.

[*α*-(2,6-Dimethylphenylthio)$_8$PcP(OMe)$_2$]$^+$[ClO$_4$]$^-$ (P-Pc): Phosphorus oxybromide (1.5 g) was added a solution of **3a** (133 mg, 83 μmol) in 2 mL of pyridine and stirred for 30 min at room temperature. After the solvent was removed in vacuo, the residue was dissolved in a solution of dichloromethane/methanol (50/50, v/v) and stirred for 30 min at room temperature. The organic layer was collected, washed with water, and solvent was removed to yield a dark green product (phosphorus Pc). The resulting solid was dissolved in dichloromethane, then $NaClO_4$ (41.5 mg, 340 μmol) was added. After the mixture was stirred for 12 h at room temperature, solvent was removed, and the residue was recrystallized from dichloromethane/n-hexane. **P-Pc** was obtained (105 mg, 75%) as a dark green powder. The UV-vis-NIR spectrum of P-Pc in chloroform solution shown in Fig. S2. ^{1}H-NMR (400 MHz, $CDCl_3$) δ (ppm): 7.36-7.33 (m, 8H), 7.29-7.28 (m, 16H), 6.87 (s, 8H), 2.52 (s, 48H), 0.36 (d, 6H). ^{13}C-NMR ($CDCl_3$) δ (ppm): 145.25, 144.33, 137.69, 130.52, 129.20, 128.52, 127.75, 21.86. ^{31}P-NMR ($CDCl_3$) δ (ppm): -180. MALDI-TOF mass calculate for $C_{98}H_{86}N_8O_2PS_8$ [M–ClO$_4$]$^+$: 1693.437; found: 1693.281.

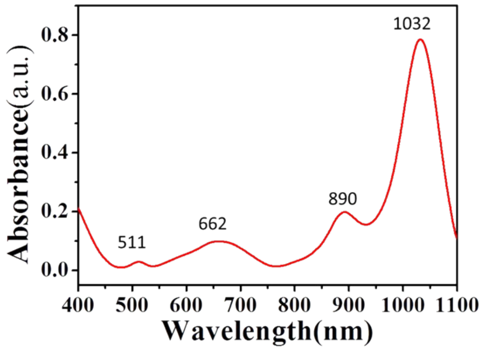

Fig. S2 UV-vis-NIR spectrum of **P-Pc** in chloroform.

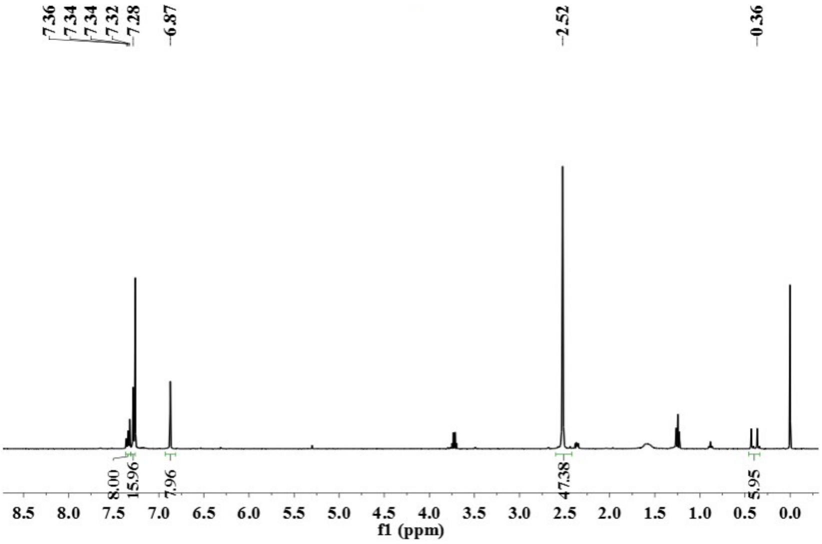

Fig. S3 ^{1}H NMR spectrum of **P-Pc** in $CDCl_3$. The signals at 7.26, 5.30, and 1.50 ppm due to residual $CHCl_3$, CH_2Cl_2, and H_2O, respectively.

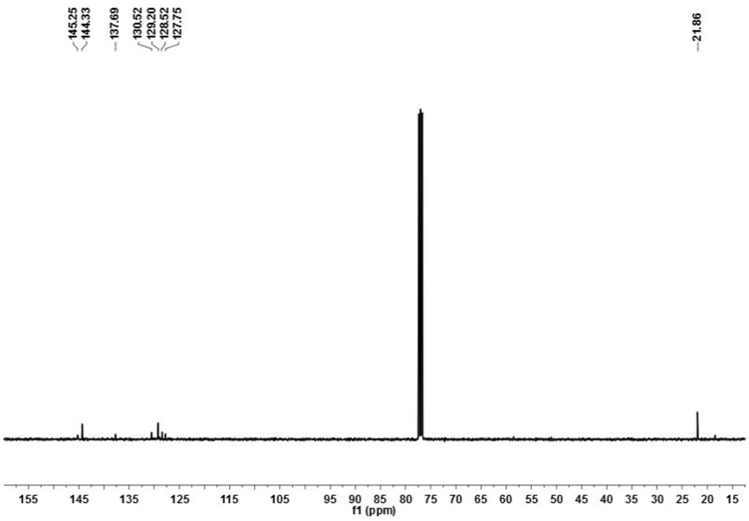

Fig. S4 ^{13}C NMR spectrum of **P-Pc** in $CDCl_3$.

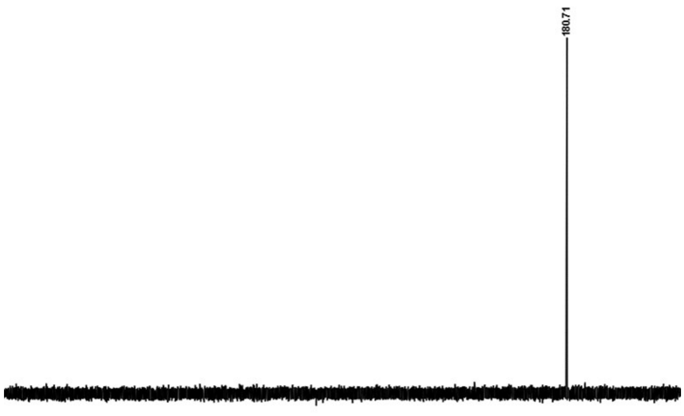

Fig. S5 ^{31}P NMR spectrum of **P-Pc** in $CDCl_3$.

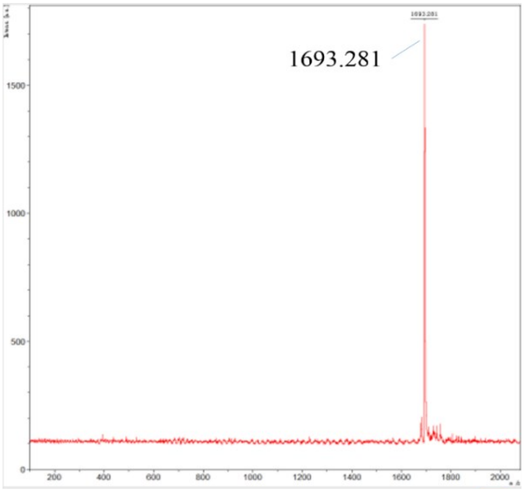

Fig. S6 MALDI-TOF mass spectrum for **P-Pc**.

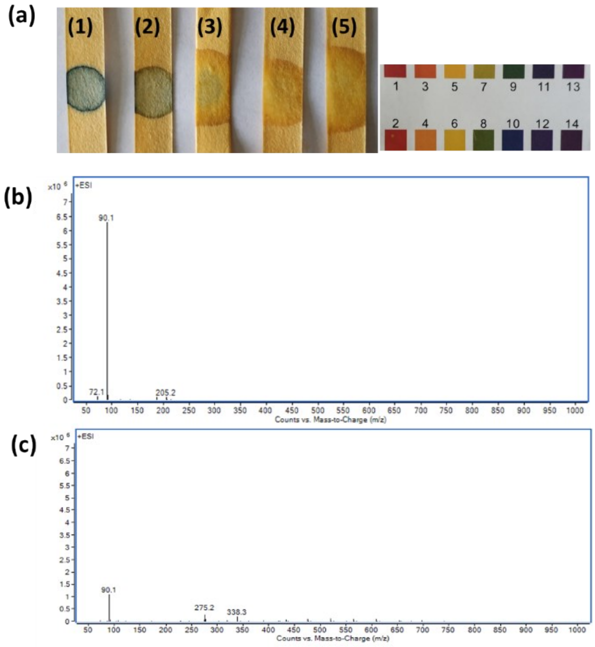

Fig. S7 (a) pH paper test for the mixed solution of DMEA:THF:H_2O (v/v/v = 1:1:20) (1) and the dialysate for the first (2), second (3), fifth (4), seven (5) times. The alkalinity of dialysate became weaker until neutral, indicating the free small organic molecules can be removed by dialysis. (b) The mass spectrum for **P-Pc-HSA** NPs solution (50 μL, 1 mL methanol) before dialysis (MS: m/z calcd for [DMEA]+ 90.1, [THF] 72.1). (c) The mass spectrum for **P-Pc-HSA** NPs solution (50 μL, 1 mL methanol) after dialysis. As shown in Fig. S7c, the content of organic molecules in **P-Pc-HSA** NPs solution was extremely low after dialysis.

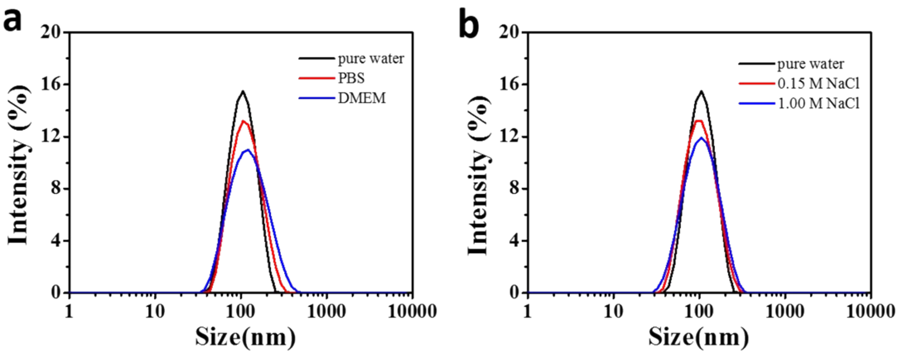

Fig. S8 Size distribution of **P-Pc-HSA** NPs in (a) water, PBS, DMEM and (b) NaCl measured by DLS.

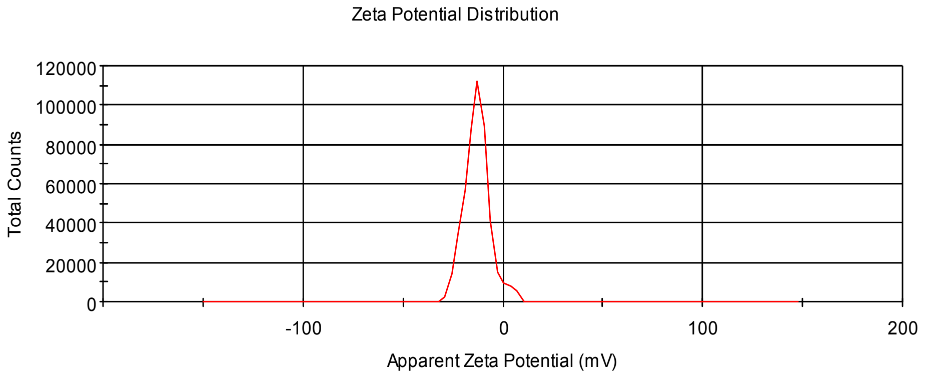

Fig. S9 ζ potential of **P-Pc-HSA** NPs in aqueous solution.

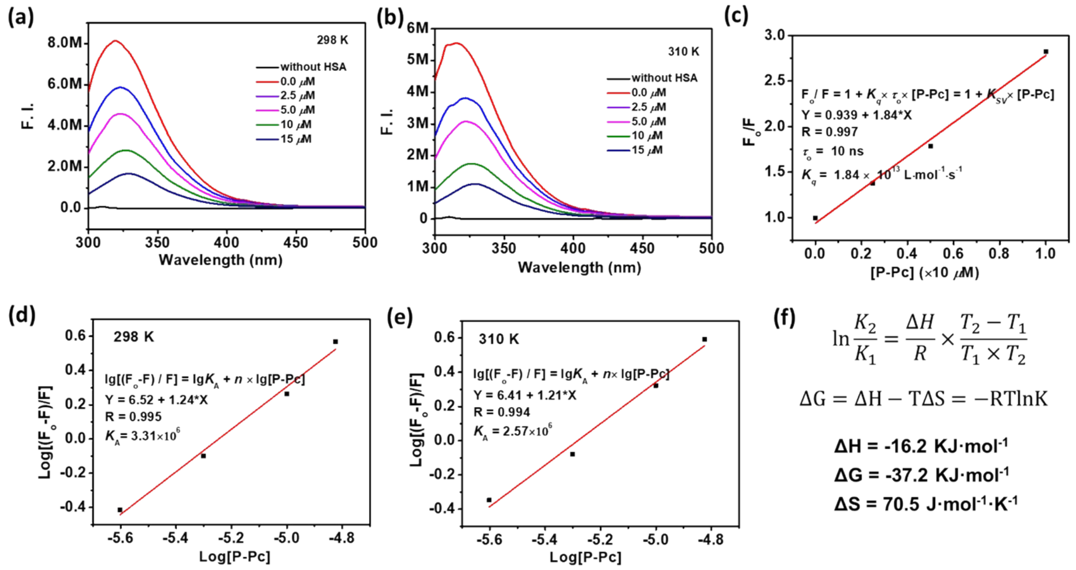

Fig. S10 Fluorescence spectra (λ_{ex} = 280 nm) of HSA (H_2O, 10 μM) at 298 K (a) and 310 K (b) quenching caused by P-Pc (DMEA/THF, v/v = 1:1, 100 μL). (c) The Stern-Volmer plot of HSA with P-Pc at 298 K. F_o/F and [P-Pc] showed good linearity relationship and the Stern−Volmer quenching constant K_{SV} was 1.84×10^5 L·mol^{-1} and the corresponding quenching rate constant K_q

was 1.84×10^{13} L·mol^{-1}·s^{-1} (τ_o = 10 ns), which is much higher than the limiting diffusion constant of the biomolecules (2.0 $\times10^{10}$ L·mol^{-1}·s^{-1}), confirming close interaction between HSA and P-Pc. Double logarithmic regression curves of HSA with P-Pc at 298 K (d) and 310 K (e). The binding constants (K_A) are higher than 10^6 and the binding stoichiometry between HSA and P-Pc is 1:1. (f) Calculation details of thermodynamic parameters of ΔH and ΔS. The thermodynamic parameters ΔH < 0 and ΔS > 0, indicating HSA interacted with P-Pc mainly by electrostatic effect.[1-3]

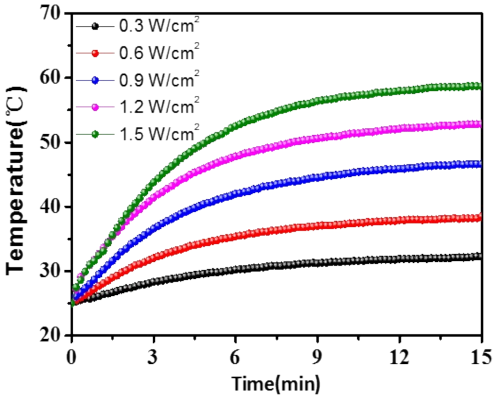

Fig. S11 Temperature increase curves of **P-Pc-HSA** NPs (72 μg/mL) exposure to the 1064 nm laser at different power density.

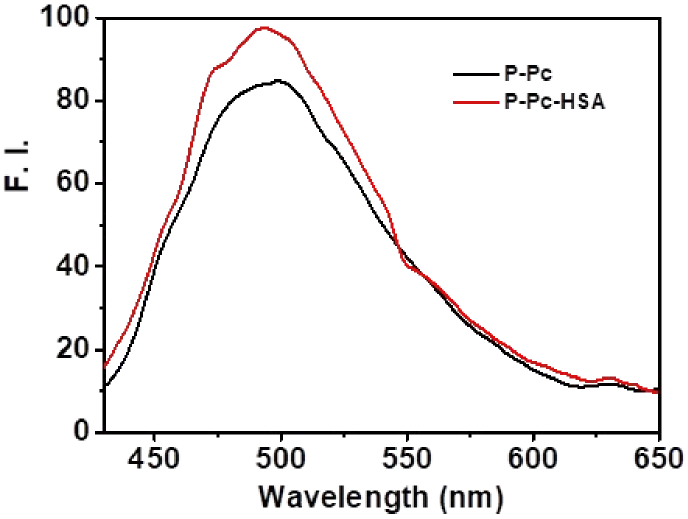

Fig. S12 Fluorescence spectra of P-Pc and **P-Pc-HSA** NPs (both at 2.5 μM, P-Pc equiv) in water upon irradiation at 405 nm.

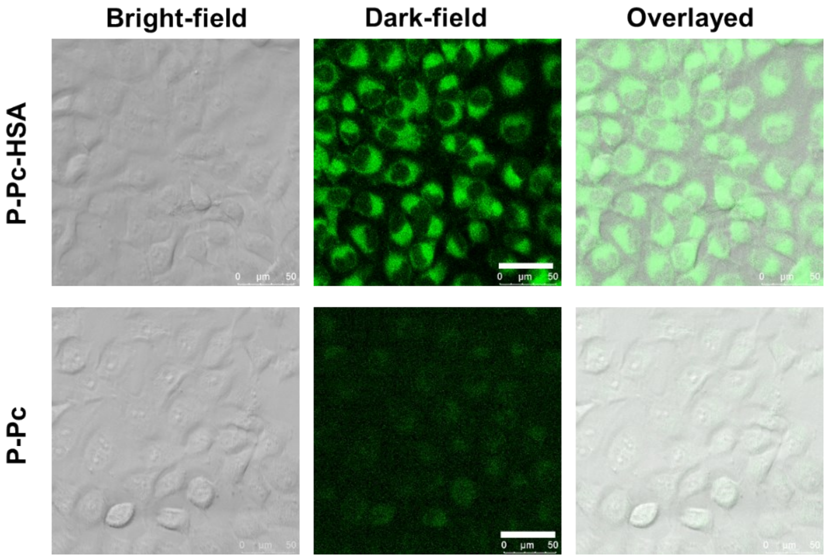

Fig. S13. *In vitro* CLSM images of MCF-7 cells treated with P-Pc and **P-Pc-HSA** NPs (both at 144 μg/mL, P-Pc equiv) for 2 h, using a green channel for phthalocyanine (λ_{ex} = 405 nm, 430-560 nm), scale bar 50 μm.

Reference

1. J. R. Lakowicz and G. Weber, *Biochemistry*, 1973, **12**, 4171-4179

2. P. D. Ross and S. Subramanian, *Biochemistry*, 1981, **20**, 3096-3102

3. X. Li, S. Yu, Y. Lee, T. Guo, N. Kwon, D. Lee, S. C. Yeom, Y. Cho, G. Kim, J. D. Huang, S. Choi, K. T. Nam and J. Yoon, *J. Am. Chem. Soc.*, 2019, **141**, 1366-1372.
